# Gender-based discrimination and unprotected receptive anal intercourse among transgender women in Brazil: A mixed methods study

**DOI:** 10.1371/journal.pone.0194306

**Published:** 2018-04-11

**Authors:** Laio Magno, Inês Dourado, Luís Augusto V. da Silva, Sandra Brignol, Leila Amorim, Sarah MacCarthy

**Affiliations:** 1 Departamento de Ciências da Vida, Universidade do Estado da Bahia, Salvador, Bahia, Brazil; 2 Instituto de Saúde Coletiva, Universidade Federal da Bahia, Salvador, Bahia, Brazil; 3 Instituto de Humanidades, Artes & Ciências Professor Milton Santos, Universidade Federal da Bahia, Salvador, Bahia, Brazil; 4 Instituto de Saúde Coletiva, Universidade Federal Fluminense, Niterói, Rio de Janeiro, Brazil; 5 Instituto de Matemática e Estatística, Universidade Federal da Bahia, Salvador, Bahia, Brazil; 6 Rand Corporation, Santa Monica, California, United States of America; University of Westminster, UNITED KINGDOM

## Abstract

**Introduction:**

Discrimination related to gender identity may directly influence vulnerability to HIV through increased exposure to unprotected receptive anal intercourse (URAI). Little is known about the relationship between gender-based discrimination (GBD) and URAI with stable partners among transgender women.

**Methods:**

This mixed-methods research began with a cross-sectional survey conducted between 2014 and 2016 with transgender women in Salvador, the capital city in one of the poorest regions in Brazil. Respondent-driven sampling was used to recruit the study population. GBD was defined through Latent Class Analysis. Additionally, 19 semi-structured interviews with participants were transcribed and analyzed through thematic content analysis.

**Results:**

URAI with stable partners was commonly reported (37.3%). GDB was positively associated with URAI among stable partners (OR = 6.47; IC 95%: 1.67–25.02). The analysis of the interviews illustrated how GBD impacted transgender women in diverse ways. Experiences with GBD perpetrated by the family often initiated a trajectory of economic vulnerability that led many to engage in survival sex work. The constant experience with GBD contributed to participants feeling an immense sense of trust with their stable partners, ultimately diminished their desire to use condoms. Further, the high frequency of GBD contributed to poor mental health overall, though some participants said engagement in transgender advocacy efforts provided a vital source of resilience and support.

**Conclusion:**

Our mixed-method study capitalizes upon the strengths of diverse data sets to produce a holistic understanding of GBD and URAI with stable partners. Furthermore, by confirming the association between greater GBD and URAI, we have demonstrated how GBD can impact condom negotiation in diverse relationships.

## Introduction

A systematic review reported that 19.0% of transgender women (TGW) are infected with HIV globally; the estimated odds of infection is more than 80 times higher than amongst adults of reproductive age in the general population [1]. The estimated prevalence for Brazil is even higher: one-third of TGW are HIV-positive, with variations depending on geographical region and data source [2–5]. In the northeast, one of the poorest areas of the country, studies have shown high rates of HIV self-reported prevalence among self-identified *travestis* and transsexual women, of 12.0% [3] and 12.7% [2].

Studies both globally and in Brazil have documented a range of factors impacting the risk for HIV infection. Behavioral risk factors such as unprotected sex [2,3,5–9] (particularly among sex workers) [5,9,10] and especially unprotected receptive anal intercourse (URAI) [11] have been shown to significantly increase rates of HIV transmission per sex act. Other behavioral risk factors including body modification practices like hormone and silicone use without medical assistance [12]; alcohol and drug use have also been shown to increase HIV risk [5,13–15].

Structural risk factors also affect vulnerability for HIV infection. Studies have shown how, for TGW in particular, constant displacement [2,16–18], social exclusion, and unfavorable socioeconomic conditions contribute to marginalization of this population [1,19–21]. Further, the relationship between discrimination and a range of negative outcomes experienced by TGW has been increasingly documented [22–25]. Gender-based discrimination (GBD) in particular, defined as any distinction, exclusion, or restriction made on the basis of one’s gender identity [26], has been highlighted as creating psychological, social, and economic vulnerability for TGW [19,27,28]. Even when compared to other vulnerable groups such as men who have sex with men, TGW consistently report higher rates of discrimination [25] and stressful psychosocial events [8].

Studies have begun to document how discrimination can increase the odds of URAI among TGW with a range of sexual partners (including sex work clients as well as casual and stable partners) [23,25]. However, to date, the relationship between discrimination and URAI with stable partners remains unclear [7,13,29]. In response to this knowledge gap, we examined the empirical association between GBD and URAI with stable partners and explored their experiences of discrimination from life histories in the largest city of Northeastern Brazil.

## Materials and methods

This investigation is part of the PopTrans’ study [30], a mixed method study that draws on two datasets–a cross-sectional survey and face-to-face semi-structured interviews. The study population consisted of *travestis* and transsexual women aged 15 years or older.

In Brazil, the terms ‘*travestis’* and ‘transsexual’ are commonly used by the communities themselves. The difference that can mark both identities would be the political and/or subjective identification as *travestis* and transsexual women. Though fluid and dependent on context, Lionço [31] argues that *travestis* and transsexual women "are subjectively constituted as a person with a gender that does not correspond to their sex assign at birth". Both terms relay differing levels of performance as a woman and claim the legitimacy of their identity beyond the binary parameters of masculine and feminine, adequacy of their physical image and their bodies using hormone therapy and silicone, desiring to be treated in the feminine and by the name with which they identified. However, *travesti* relay a sexual ambiguity or duplicity with the maintenance of the penis in the way to reaffirm their identity. Transsexual women can relay different degrees of transitioning usually seeking the adequacy of their physical image and their bodies but not always seek sex reassignment surgery. Furthermore, there is a transit between these identities, not being fixed or isolated, but always in dispute, negotiation, in constant interaction and movement [30,32]. Therefore, to acknowledge the Brazilian context, we use the term *travestis* and transsexual women (TrTW) in this article to reference our study participants and use TGW to refer to transgender women more broadly in the international literature.

All participants in the study lived in Salvador or in the metropolitan region throughout the study period. Salvador, capital of Bahia State, is Brazil’s fourth largest–and one of its lowest-income–cities. All data collection occurred between September 2014 and April 2016. The research project was approved by the Research Ethics Committee of the State Health Department of Bahia, and all participants provided written informed consent. For participants between 15–17 years old, an assenting consent form was signed by participants and a consent form was signed by a Youth-NGO in accordance with Research Ethics Guidelines in Brazil.

### Quantitative data

The study team used respondent driven sampling (RDS) to recruit study participants. RDS is a chain-link sampling method that begins with “seeds”- a convenience sample of members of the target population chosen by the researchers [33,34]. We conducted formative research to identify 10 TrTW “seeds” who had relatively large social contact networks with a diverse age range and socioeconomic makeup. Each participant received three coupons to recruit other participants to the study. The best sample size is achieved when the recruitment reaches its limits given the amount of time for data collection. It is worth noting that, based on standards established in the peer-reviewed literature on RDS [34], our data collection was very long (for two years) and we reached saturation with sample size of 127 TrTW. Participants received an initial incentive of R$ (Brazilian Real) 30.00 US$10.60, and an incentive of R$ (Brazilian Real) 30.00 (US$10.60) for each of their recruits who completed the survey. Coded coupons and coupon manager software were used to minimize duplications. Survey data were collected by oral interviews, and responses were recorded using a tablet. Interviews took place in three rooms of a building located in Salvador’s historical center, a known cruising area for TrTW and easily accessible by public transportation.

#### Main exposure variable

Latent Class Analysis (LCA) was used to define patterns of GBD based on the variables from the survey question defined: “have you suffered from any of the following experiences because of your gender identity? (yes; no) (1) discrimination from private security guards (2) discrimination by family members (3) discrimination by friends (4) discrimination by neighbors, (5) police violence, (6) physical violence and (7) verbal violence. LCA is a statistical method used to classify similar individuals in latent (unobserved) classes, when a set of observed categorical variables are correlated to each other. The main objective of this analysis for our data is to classify a heterogeneous group of TrTW into subpopulations according to patterns of GBD. The subpopulations, represented by the latent classes, will be characterized based on their item response probabilities [35].

#### Outcome variable

URAI with stable partners was determined by responses to the following question: “Do you often use condoms with your stable partners (boyfriends or husbands) during anal sex when penetrated by your partner?” This question did not refer to a specific period of time and it was answered only by TrTW who had stable partners. It had the following options of response: “always, most of the time, rarely, never.” Since the sample size was not sufficient to use a multinomial logit analysis, the responses were dichotomized as “Always, most of the time, or rarely used condoms”, and “never used condoms”.

#### Other key variables

URAI with casual partners and clients was determined by responses to the following question: “Do you often use a condom with your casual partners (or with clients) during anal sex when being penetrated by your partner?” Responses were categorized in “always, most of the time, or rarely used condoms” and “never used condoms”. Researchers identified a set of variables as potential confounders of the main association (GBD and URAI with stable partners). Variables included: gender identity (self-identified as *travesti or* transsexual woman); age (≤ twenty years, twenty-one to thirty-four years, and ≥ thirty-five years); years of schooling (≤8 years, 8 to 12 years, ≥ 12 years); self-reported skin color (white, black, *pardo*-someone from a mixture of skin color, or brown); marital status (married or live with a partner and single); individual income—receiving minimum wage of R$788 Brazilian Real per month = $262 USD based on the exchange rate at the time of the data collection—this is how monthly salaries are counted in Brazil and allows for comparison with other national studies. Further, income was dichotomized because the sample size was insufficient for multinomial logit analysis; history of sex work (yes or no); and history of forced sex (yes or no). These variables were selected because they were associated with both the exposure and the outcome and were also emphasized in related peer-reviewed articles.

The presence of depression symptoms was defined by scores from the Patient Health Questionnaire (PHQ-9), validated for the adult Brazilian population to screen for depression symptoms in primary health care [36–38]. The maximum score for the PHQ-9 is 27 points. Categories were: absence of depression symptoms (0 to 9 points), light depression symptoms (10 to 14 points), moderate depression symptoms (15 to 19 points) and severe depression symptoms (20 to 27 points) [38]. Subsequently we dichotomized this variable as “absence of depression symptoms” and “mild, moderate, or severe depression symptoms.” It was dichotomized as we wanted to compare TrTW who had at least one depression symptoms with those who had none and because the sample size was not sufficient for a multinomial logit analysis. Partner trust was defined by responses to the following question: “do you think it is unnecessary to use a condom when you trust your partner?” Responses were dichotomized as “yes” or “no”.

#### Quantitative analysis

Social network size (degree) was measured from the number of TrTW each participant knew within the target population. The questions that identified social network size were: “How many *travestis* and transsexual women do you know by name and know you by name in the municipality of Salvador?” and “Of the travestis and transsexual women that you know, how many would you invite to participate in this research?”. Then we used the individual’s probability of selection a posteriori proportional to the TrTW’s social network size to calculate RDS sampling weights in order to adjust for the increased probability that a high degree TrTW would preferentially be sampled [33,39].

We used the program RDS Analyst 0.42 to calculate the weights based on the RDS-II estimator [40]. We used the parameters of LCA to describe the latent classes for the final model selected. To select the best model, we used the Bayesian Information Criterion (BIC) as well as the Akaike Information Criterion (AIC), with lower values indicating better adjustment. We fit and compared models with varying number of latent classes (from 2 to 6). We also used the entropy to summarize the uncertainty of a posteriori classification and provides an indication of discrimination of the classes defined by the model (the closer to 1 the better) [41]. The choice of model was based on the combination of statistical criteria, parsimony and interpretability [35]. LCA was performed on Mplus 5.21 software [42]. The latent variable GBD and the weighting of the RDS were transferred to the database for descriptive and multivariate analyses using STATA 12.1 (StataCorp, College Station, TX, USA). We used multiple logistic regression to estimate the effects of the main exposure variable on URAI with stable partners, allowing the assessment of confounding. For variable selection to be maintained in the final model for the URAI, the forward procedure based on the literature review was considered.

### Qualitative data

The qualitative data was not intended to establish a causal pathway between GBD and URAI, but instead to contextualize concrete situations of discrimination and URAI from in-depth interviews. To achieve this, we invited a convenience sampling of 19 TrTW participants from the quantitative survey to concurrently participate in the qualitative arm of the study. The selection process took into consideration the same inclusion criteria used for the survey and the sociodemographic diversity observed by the interviewers in their fieldwork diaries. Individuals were asked to participate based on the following characteristics: age; gender identity; educational attainment; skin color; income; and marital status. Narratives about GBD included a discussion of the respondent’s life history, experiences of discrimination and violence (verbal, physical and sexual), access to health care, and body modification procedures, as well as sexual behavior. We developed and extensively field tested semi-structured interview scripts, which included open-ended questions. All interviews were conducted individually and ranged from 44 minutes to 2 hours and 30 minutes. All participants provided written informed consent. To preserve confidentiality, we conducted all interviews in a room at the survey headquarters.

#### Qualitative analysis

We analyzed transcripts and related field notes, and one of the authors (LM) identified deductive codes based on a review of consistent themes that emerged from the literature (e.g. use of condoms; sex work; sexual violence; physical and verbal aggression). Next, we used inductive codes to identify new factors in the transcripts that may not have been highlighted by the existing literature (e.g., GBD perpetrated by friends and family; GBD and mental health; URAI with stable partners). We conducted content analysis, and as categories emerged, we identified exemplars to further illuminate the key themes emerging from each interview. Data were reviewed and discussed by two authors (LM and LAVS), and key themes were discussed with the entire study team.

## Results

### Quantitative results

Most participants reported more consistent condoms use with casual partners (95.2% CI: 89.67–97.89) and with clients of sex work (99.7% CI: 98.1–99.9). The proportion of TrTW that never used condoms with stable partners was 37.3% (95% CI: 28.3–46.2). Most TrTW (81.1% CI: 66.0–90.4) reported sexual intercourse only with men, fewer reported having sexual intercourse with men and women (17.8% CI: 8.6–33.1) or intercourse with women and other TrTW (1% CI: 0.3–3.4) (Table 1).

**Table 1:** Descriptive statistics of the study population.

Variables	N	% Crude	% Weighted^a^
**Sociodemographics**			
**Gender identity**			
Transsexual woman	67	52.8	53.8
*Travesti*	60	47.2	46.2
**Age**			
≤ 20 years	39	30.7	34.8
21–34 years	54	42.5	43.5
≥ 35 years	34	26.8	21.7
**Years of schooling**			
≤ 8 years	49	38.6	26.8
8–12 years	66	52	64.5
≥ 12 years	12	9.4	8.7
**Skin Color**			
White	24	18.9	19.6
Black	38	29.9	28.6
*Pardo* (Brown)	65	51.2	51.8
**Marital status**			
Married or live with a partner	35	27.6	36.6
Single	92	72.4	63.4
**Monthly Income (minimum wage = US$262)**			
≤ **US$262**	45	35.4	39.1
**US$262$-524**	23	18.1	13.8
≥ **US$524**	59	46.5	47.1
**History of sex work**			
No	16	12.6	7.9
Yes	111	87.4	92.1
**Blackmailed or extorted for money**			
No	89	70.1	72.9
Yes	38	29.9	27.1
**Legal name change on government ID card**			
No	114	96.6	98.3
Yes	4	3.4	1.7
**Legal name changed on health service ID card**			
No	101	87.8	87
Yes	14	12.2	13
**Ever experienced discrimination while seekinghealth services**			
No	63	59.4	51.6
Yes	43	40.6	48.4
**Sexual Behavior Variables**:			
**URAI with stable partners**			
Always, most of the time, or rarely used condoms	83	71.6	62.7
Never used condoms	33	28.4	37.3
**URAI with causal partners**			
Always, most of the time, or rarely used condoms	119	93.7	95.2
Never used condoms	8	6.3	4.8
**URAI with sex work clients**			
Always, most of the time, rarely or used condoms	110	99.1	99.7
Never used condoms	1	0.9	0.3
**Sexual intercourse in last 6 months**			
With men only	104	83.9	81.1
With men and women	16	12.9	17.8
With *travestis* and transsexual women	4	3.2	1.1

^a^Weighted by RDS-II estimator.

We analyzed models with 2 to 6 latent classes. The model chosen to describe GBD was the one with two latent classes (Table 2). TrTW classified in class 1 (n = 53) denominated “high GBD,” reported high rates for all the LCA variables: discrimination from private security guards (100%), discrimination by family members (65%), discrimination by friends (53%), discrimination by neighbors (78%), police violence (94%), physical violence (86%), and verbal violence (95%). TrTW classified in class 2 (n = 74) denominated “lower GBD” reported lower rates for most of the LCA variables: discrimination from private security guards (15%), discrimination by family members (54%), discrimination by friends (34%), discrimination by neighbors (58%), police violence (7%), physical violence (40%), and verbal violence (81%) (Table 3).

**Table 2:** Criteria for comparing models with different number of classes for gender-based discrimination using latent class analysis (n = 127).

Criteria	2 classes	3 classes	4 classes	5 classes	6 classes
AIC^a^	941.141	936.526	938.159	935.809	945.395
BIC^b^	983.804	1.001.942	1.026.329	1.046.732	1.079.072
Sample-Size Adjusted BIC^b^	936.368	929.206	928.294	923.398	930.438
Entropy	0.878	0.868	0.836	0.800	0.848

^a^AIC: Akaike Information Criterion.

^b^BIC: Bayesian Information Criterion.

**Table 3:** Latent class analysis for gender-based discrimination for two classes (n = 127).

Variables	Overall%	Gender-based discrimination	
		High % (N = 53;41.5%)	Low % (N = 74;58.5%)
Discrimination by private security guards	54.7	100.0	15.2
Discrimination by family	59.4	65.4	54.2
Discrimination by friends	42.5	52.6	33.6
Discrimination by neighbors	67.0	77.5	57.8
Any police violence	47.2	94.1	7.2
Physical violence	59.1	85.5	40.3
Verbal violence	86.6	94.9	80.7

There was a positive association between GBD and URAI with stable partners (OR = 4.55; 95% CI: 1.29–15.98). This association was even stronger after adjusting for age, income, skin color, years of education, history of forced sex, and gender identity (OR = 6.47; 95% CI: 1.67–25.02) (Table 4).

**Table 4:** Odds ratio between gender-based discrimination and URAI with stable partners, estimated by logistic regression models (n = 116).

Models	OR^a^ (CI 95%)	P-value
GBD (crude)	4.55 (1.29–15.98)	0.018
GBD (adjusted by age)	4.82 (1.27–18.29)	0.021
GBD (adjusted by age and income)	4.91 (1.25–19.24)	0.023
GBD (adjusted by age, income, and skin color)	5.11 (1.27–20.55)	0.022
GBD (adjusted by age, income, skin color, and education)	4.97 (1.23–19.94)	0.024
GBD (adjusted by age, income, skin color, education, and experience with history of forced sex)	6.24 (1.48–26.30)	0.014
GBD (adjusted by age, income, skin color, education, history of forced sex and gender identity)	6.47 (1.67–25.02)	0.007

^a^Weighted estimate by RDS-II estimator.

Among TrTW with mild, moderate, or severe depression symptoms, the association between GDB and URAI with stable partners was strong (OR = 44.4). Further, amongst those who trusted their partner, the association between GBD and URAI with stable partners was also very strong (OR = 25.4). However, due to small sample size further analysis was not conducted as the estimates would be quite imprecise.

### Qualitative results

The qualitative results illustrated different ways in which GBD impacted a range of relationships for TrTW, including their friends and family, sex work clients, and stable partners. For example, although participants reported experiencing GBD from a variety of people, it appeared most hurtful when perpetrated by family and friends. GBD was common in sex work and often accompanied by episodes of sexual violence and URAI. Constant experiences with GBD appeared to augment the trust that TrTW had in their stable partners and diminished their desire to use condoms. Another consequence of constant GBD was poor mental health, though engagement in efforts to advocate for transgender rights locally provided a source of resilience for several participants.

#### Experiences with GBD were most hurtful when perpetrated by friends and family

Both the quantitative and qualitative analyses found that experiences with GBD were commonly perpetrated by neighbors; however, the semi-structured interviews suggested that the impact of GBD perpetrated by family and friends was far greater. One participant reported intense emotional suffering following a friend’s rejection after the participant had started the process of bodily transformation and affirmation of identity.

People say that they accept it, so why don’t they invite me to their house? After I am dressed as a trans person? After I assumed my identity, I was never again called by my friends to go out, to go to their houses, to the beach or the cinema

(*27 years old, transsexual woman*)

Since I was a child I have always been girlish and grew up like this (…) I wanted to get away from my mother’s presence, but she was the one to kick me out (…) Since I was 14 years old I have lived my life apart from my family

(*61 years old, travesti*)

There was a time when I realized and thought to myself: I want to be a woman. I want to internalize and externalize the woman inside me. I no longer supported my life as half man and as half woman because my family did not accept who I was. And at age 15 I engaged in prostitution because I was expelled from my parent´s home. And I started looking for clients in the streets of Salvador

(*27 years old, transsexual woman*).

Due to the discrimination suffered within their families, many TrTW left home, initiating a trajectory of displacement that increased their economic vulnerability and pushed many participants into sex work.

#### GBD was common in sex work and often accompanied by episodes of sexual violence and URAI

The types of aggression experienced by TrTW were highly diverse, ranging from symbolic violence, verbal aggression (e.g., swearing, jokes), physical aggression (e.g., fights, histories of homicide), and especially sexual violence (e.g., rape). The interviews show a banalization of this violence, which the perpetrators justified by the bodily performance or feminine identity of TrTW:

*A friend was murdered (…) She had mannerisms; similarly, she used clothes, switching between masculine and feminine. Her hair was much longer than mine, she had a feminine face. (…) There was an argument with a homophobe. (…) During the argument, he left and when he returned, he came back armed and shot her three times*.

(*49 years old, transsexual woman*)

URAI due to rape was common in the context of sex work. One participant summarized why by saying, “…the client takes advantage of [my] vulnerability and asks for sex without a condom, and offers me more money for sex without a condom” *(49 years old*, *transsexual)*. Other experiences relayed:

*I was raped … I was forced to have sex with a drug user who put a knife to my stomach*. *He came up from behind*, *put a blade to my neck and said “let’s go*, *I’ll stick this in your neck*, *don’t run*, *you whore…” And then I descended to the sand of the beach*, *he ordered me down and fucked me*. *He came in me… I [also] suffered assault from clients who raped me*. *With the risk of getting HIV*, *[I was forced to have] sex with a gun to my head*.

(*27 years old, transsexual woman*)

…*he put a gun to my head and then I shit all over so they did not want to do anything*, *and even then they raped me*, *all dirty*, *no condom and nothing* … *I got there all traumatized*. *All full of shit*, *all full of sperm in the body*.

(*56 years old, travesti*)

#### Constant experiences with GBD contributed to infrequent condom use with stable partners

Condom use was infrequent among stable partners, and participants said the trust they had in their relationship negated the importance of wearing a condom. Importantly, respondents suggested that since stable partners often protected them–which many said was of paramount importance given constant experiences with GBD—they did not feel the need to protect themselves against HIV when having sex with their stable partners.

*I mess with him (her husband) without a condom. We’ve been together for 5 years. And … with him I’m safe. I think it’s because he is defending me*.

(*27 years old, transsexual woman*)

*If it’s a new boyfriend and I do not know him yet, then no sex without a condom. First I need to trust him, talk to him, see his situation, his health status. Gradually I try to see if he is clean. But, at least I do a blowjob without a condom. When I met my husband I engaged in sex without a condom*.

(*26 years, transsexual woman*)

#### Constant experiences with GBD contributed to poor mental health for many participants

The interviewee quoted here reported intense depression symptoms:

*I thought about killing myself. I didn’t accept who I was. Do you know why I didn’t accept myself? I don’t have a family that accepts me. I don’t have anybody. For me there was no pleasure in the world. I ended up taking medication to try to kill myself*.

(*27 years old, transsexual woman*).

*Sometimes it happens in the head “Oh, I wanted it to end all this.” Depression, (…) The lack of the family, that I am linked to the family, to talk. We have a very nightly life and we ended up not talking much in the house*.

(*40 years old, travesti*).

However, reports of overcoming and resisting GBD were also common. Two participants in particular referenced their engagement in activism to advance transgender rights as a critical source of support for them.

In sum, these interviews showed that GBD impacted the participants in our study in diverse ways. Experiences with GBD perpetrated by the family often launched a trajectory of economic vulnerability that led many to engage in survival sex work. Participants reported that this sense of trust diminished their desire to use condoms with their stable partners. Finally, the constant experiences with GBD contributed to poor mental health overall, though some participants still reported resilience through their engagement with transgender advocacy work.

## Discussion

Participants in this study reported high levels of discrimination from police, family, friends, and neighbors. Further, GBD was often accompanied by violence. This pattern is not unique to Brazil; numerous studies globally have shown how TGW report frequent experiences with physical [8,43–47], verbal [8,43–47], symbolic [44,48,49], emotional [8], and sexual violence [16,43,50–54]. The mixed-methods data show that GBD is a complex phenomenon in Brazilian society: the quantitative data showed that it can influence the use of condoms in sex with stable sexual partners, and the qualitative data suggested that the trust that TGW feel for their stable partners can also increase their willingness to have sex without a condom.

Several aspects of Brazilian culture may contribute to the frequent experiences with GBD. For example, “*machismo*” (male chauvinism), misogyny, and patriarchy [55] are dominant traits in Brazilian society. In many situations, these traits contribute to behavior that subjects TrTW to stigma, discrimination, and violence. Although LGBT organizations in Brazil are well-established, especially with the advent of the HIV/AIDS epidemic, Brazil has still not developed legal mechanisms to respond to discrimination against TrTW [56]. Furthermore, current religious, political, and other organized groups in Brazil are taking concrete action to diminish existing protections for TrTW [57,58]. This is hugely problematic given recent estimates showing that Brazil is home to the highest number of murders among TGW in the world [59,60].

TrTW found GBD perpetrated by family members especially painful, and studies have documented how discrimination in the family can increase the vulnerability of TrTW to HIV infection. As echoed in our qualitative interviews, for many TrTW, GBD perpetrated by family is the first step toward social exclusion, followed by expulsion from their homes, high levels of school dropout, and initiation of sex work. Similarly, studies in Brazil [47], Mexico [8], and the US [29] found that experiences of GBD within the family often served as the genesis of TGW leaving their home and struggling to find viable economic opportunities. Often survival sex work was one of the only available options for earning money.

The quantitative analysis revealed that the practice of URAI with stable partners was common among TrTW in the sample. Previous studies have documented the relationship between URAI and sexual partners more generally, but not specifically with stable partners. For example, a study conducted with TGW in three African countries showed an association between different acts of discrimination and URAI, though the authors did not indicate the type of sexual partner [25]. A US-based study showed that among TGW ages 18–25, those reporting higher levels of exposure to transphobia (e.g., discrimination of individuals who do not conform with the traditional gender norms), were three times more likely to report URAI [23].

We build on these findings by exploring the relationship between URAI and stable partners. The qualitative data from our study suggest that the trust TrTW felt for their stable partners contributed substantially to their willingness to have sex without a condom. Specifically, the constant experiences with GBD meant that when TrTW recognized the possibility of a longer lasting connection, or had some indication of trust, it diminished their perceived need to use a condom. A qualitative study of TGW in the US [29] reported similar findings, describing how participants negotiated condom use differently between clients and relationships. Condom use was used more often in sex work, which was understood as a business with well-defined rules, one of which was nonnegotiable condom use. Sex with stable partners was imbued with personal and emotional significance and was less influenced by the importance of practicing safer sex.

Other studies have suggested that URAI can also serve as a way to affirm the femininity of TGW. The figure of the “husband” was highly desired by the *travesti*—it was a form of affirmation of their feminine status and made them feel like a “woman.” Further, the stable partner needed to be a “man,” which often meant he played the “active” role during anal sex. In contrast, during sex work, the sexual role varied depending on the client [61]. Thus the potential to consistently play the more feminine role during sex with their stable partners may have increased the frequency of URAI among TrTW, compared to more vary sexual positioning with sex work clients. We did not explore the potential for URAI to affirm TrTW femininity. Future studies should aim to disentangle how these complex understandings of trust–and its impact on risk perception–may impact HIV risk among TGW in particular. Furthermore, studies should collect information on HIV serostatus for stable partners, as this may influence URAI.

We also found some evidence that depression symptoms might modify the relationship between GBD and URAI among stable partners, but we did not have enough power to make conclusive inferences. Studies have shown that discrimination can be harmful to the mental health of TGW [62,63] and the relationship between mental health and HIV risk has been clearly documented. For example, a survey among TGW in the US showed that the chance of URAI was almost three times higher among participants with low self-esteem [64]. Corroborating these findings, a longitudinal study in the US showed that the combination of GBD and depression symptoms was an important predictor for URAI [65]. Another study with Asian Pacific Islander TGW in the US reported a positive association between URAI and previous attempted suicide [66]. Finally, a qualitative study with TGW in India also showed that interviewees with low self-esteem said that they did not use condoms with their husbands, mainly because the condom presented a barrier to intimacy [7].

We have documented the complex relationship between GBD and URAI with attention to the unique roles of trust in partner and mental health. Going forward, research, programs, and policies need to address the HIV risk introduced by URAI. For example, the meaningful participation of TGW in the research process is critical to make sure that the right questions are asked, the appropriate methods are applied, and the interpretation of research findings are grounded. With respect to programs, there is ample evidence illustrating how GBD affects access to general health services in Brazil [67,68] and HIV counseling and testing [8,13,67] as well as other prevention services in particular [53,69]. Concrete steps are needed, such as training providers responsible for transgender-specific health care more broadly (e.g., knowing the appropriate dosage of hormones) as well as ensuring culturally competent care (e.g., understanding the importance of appropriate pronoun use), in addition to ensuring providers are prepared to screen TGW for experiences with GBD and connect them to the appropriate resources. Further, careful consideration is needed regarding how programs can effectively message the importance of safer sex practices, especially with a range of different sexual partners and with whom TGW may have varying levels of trust. Many individuals, regardless of their gender identity, relinquish condom use once trust is established in a relationship; consequently, a call for 100% condom use among TGW specifically may fall on deaf ears. Therefore, a more nuanced approach that continues to emphasize the benefits of condom use, while also introducing additional prevention strategies such as Pre-Exposure Prophylaxis (PrEP), will prove critical to minimizing the substantial risk of HIV faced daily by TGW both in Brazil and beyond.

### Limitations and strengths

This study has several limitations. For example, the cross-sectional design did not allow us to verify the temporal relations between exposure and outcome. Additionally, the use of RDS did not support generalizing results for the *travestis* and transsexual women populations of Salvador because the probabilistic sample generated a possible selection bias by attracting people with lower economic status. Another limitation is the use of the latent variable GBD as an observed variable (without incorporating measurement errors) in the logistic regression models. The literature suggests that this type of approach, also termed “naïve”, can result in underestimation of the effects [70]. However, more sophisticated methods for incorporating measurement errors generally require larger samples. Despite these limitations, our mixed-method study capitalizes upon the strengths of diverse data sets to produce a holistic understanding of GBD and URAI with stable partners. Furthermore, by confirming the association between greater GBD and URAI, we have demonstrated how GBD can impact condom negotiation in diverse relationships.

## Conclusions

Consistent with the national and global literature, many TrTW in our sample reported GBD. We also showed that depression symptoms as well as trust in stable relationships may further modify the relationship between GBD and URAI. Therefore, additional prevention strategies should be considered—e.g., frequent HIV testing and consistent and correct use of PrEP and Post-Exposure Prophylaxis. Finally, policies are needed to address GBD routinely faced by TGW. To date, Brazil has still not established any legal mechanisms for combating GBD, or suggested effective policies to promote social and cultural equality amongst diverse gender identities more generally [71]. Most immediately, anti-discrimination policies are needed; once available, they must be enforced. Further, guidelines to support the effective implementation of these policies (e.g., explaining how schools can create a gender-inclusive environment) will be critical to ensuring that the pervasive GBD experienced by TGW is not only diminished, but ultimately eliminated.

## Supporting information

S1 File Survey questionnaire. (RAR)
